# A simple high performance liquid chromatography method for determination of rebamipide in rat urine

**DOI:** 10.1016/j.mex.2014.06.002

**Published:** 2014-06-30

**Authors:** Dustin L. Cooper, Sam Harirforoosh

**Affiliations:** Department of Pharmaceutical Sciences, Gatton College of Pharmacy, East Tennessee State University, Johnson City, TN 37614, United States

**Keywords:** Rebamipide, HPLC, Extraction, Fluorescence, Urine, Ofloxacin

## Abstract

Rebamipide is a mucoprotective agent commonly used to prevent nonsteriodal anti-inflammatory drug-induced gastrointenstinal side effects [1]. Human plasma and urine analysis of rebamipide utilizing high performance liquid chromatography (HPLC) have been reported [2]. Recently, we reported on the plasma levels of rebamipide in presense or absence of celecoxib or diclofenac in rats [3] using a modified HPLC method of detection developed by Jeoung *et al*. [4]. To tailor the method towards use in urinary rebamipide extraction and analysis, the following modifications were made:

• To compensate for high concentrations of rebamipide found in urine, a new rebamipide stock solution was prepared with a final concentration of 50,000 ng/mL.
• Rat urine calibration standards were obtained within the range of 50–1000 ng/mL and 1000–50,000 ng/mL.
• Plasma samples were replaced with urine samples.

## Graphical abstract

**Figure:**
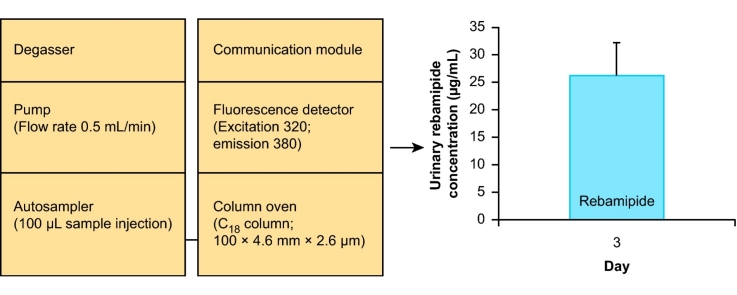

## Method details

Rebamipide urine analysis was carried out using samples obtained from a previous study [5]. In the study, male Sprague-Dawley rats were dosed with rebamipide (30 mg/kg) twice daily for two days via gastric intubation. On day three, animals received one dose of rebamipide in the morning then were transferred to metabolic cages for a 12 h urine collection period.

Analysis of rebamipide concentrations in the urine involved preparation of rebamipide stock solution, development of low and high linear standard calibration curves to compensate for altered rebamipide concentrations noted in urine samples, use of acetonitrile and hydrochloric acid for urinary rebamipide extraction, and HPLC analysis of rebamipide using a Shimadzu HPLC system.

HPLC analysis was carried out using a C_18_ analytical column with a flow rate of 0.5 mL/min and a fluorescent detector set at an excitation wavelength of 320 nm with an emission wavelength of 380 nm.

### Materials

Rebamipide powder (Tokyo Chemical Industry CO., Tokyo, Japan) (Cat. R0085). Ofloxacin powder (Sigma–Aldrich, St. Louis, MO, USA) (Cat. PHR1168-1G) HPLC grade methanol (Fisher Scientific, Pittsburg, PA, USA) (Cat. A9984). HPLC grade acetonitrile (Fisher Scientific, Pittsburg, PA, USA) (Cat. BP2405SK-4) 36% hydrochloric acid (Sigma–Aldrich, St. Louis, MO, USA) (Cat. 258148) HPLC grade water (Fisher Scientific, Pittsburg, PA, USA) (Cat. W5-4). HPLC grade glacial acetic acid (Fisher Scientific, Pittsburg, PA, USA) (Cat. A35-500). Drug free rat urine samples. Experimental urine samples from rats dosed with rebamipide.

### Preparation of stock solutions

Rebamipide stock solution was prepared by dissolving 10 mg rebamipide powder in 200 mL methanol for a final concentration of 50,000 ng/mL. Ofloxacin was used as an internal standard (IS). Ofloxacin stock solution was prepared by dissolving 100 μg ofloxacin powder in 200 mL acetonitrile. Standard solution was then vortex mixed until fully dissolved for a final concentration of 500 ng/mL.

### Development of calibration curves

**Fig. 1:**
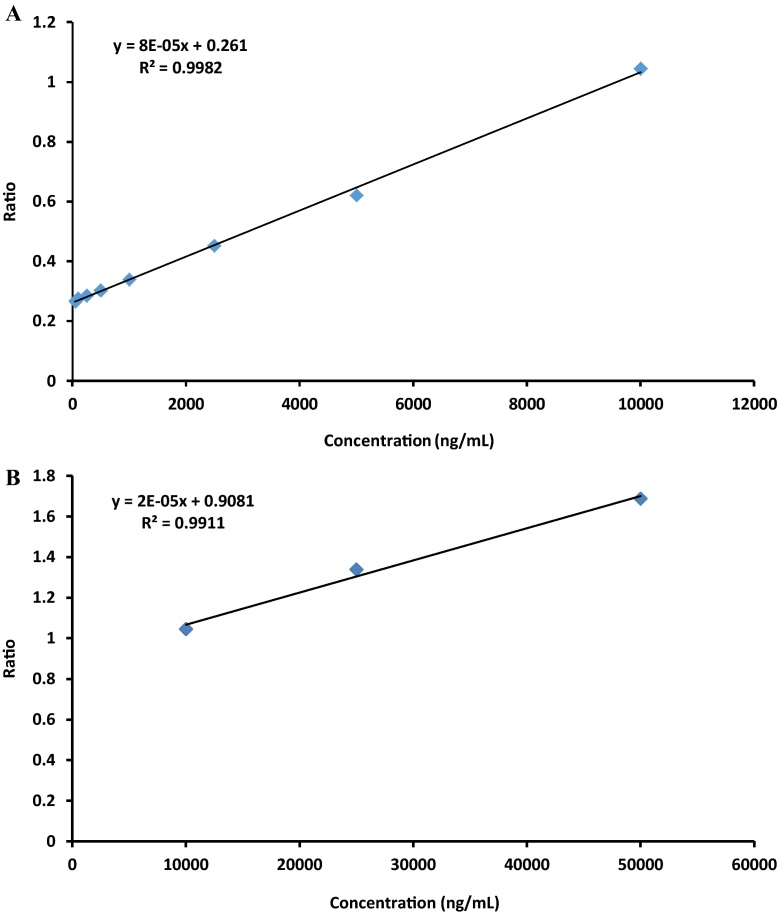
Average standard calibration curve (*n* = 3) for urinary rebamipide determination at (A) 50–10,000 ng/mL and (B) 10,000–50,000 ng/mL. Each point represents urinary rebamipide detection under fluorescence HPLC set at 320 excitation and 380 emission. Also shown are the slope and *y*-intercept values used for determination of sample concentrations.

To develop new standard calibration curves based on higher rebamipide stock concentrations, the following steps were followed:

1. Drug free rat urine (100 μL) was placed in a clean glass tube.
2. Samples were spiked with 100 μL IS.
3. Using methanol, rebamipide stock solution was serial diluted to 50,000, 25,000, 10,000, 5,000, 2,500, 1,000, 500, 250, 100, and 50 ng/mL.
  a. From the dilutions, two calibration curves with different concentration ranges were constructed.
    i. A low range calibration curve was prepared to include 50, 100, 250, 500, 1000, 2500, 5000, and 10,000 ng/mL rebamipide (Fig. 1A).
    ii. A high range calibration curve was prepared to include 10,000, 25,000, and 50,000 ng/mL (Fig. 1B).
4. Rat urine was spiked with 100 μL rebamipide followed by the addition of 20 μL of 36% hydrochloric acid.
5. Acetonitrile (2 mL) was added and solutions were vortex mixed for 1 min.
6. After vortex mixing, solutions were centrifuged for 20 min at 5000 × *g*.
7. Supernatant was transferred to a clean glass tube and evaporated to dryness using a CentriVap (Lab Conoco, MO, USA) concentrator set at 50 °C.
8. Following evaporation, samples were reconstituted in 200 μL HPLC mobile phase.
9. Re-suspended solutions (115 μL) were transferred to clean injection vials and 100 μL was injected into HPLC for analysis.
10. The chromatogram was quantified based on area ratios of rebamipide to ofloxacin.

### Sample extraction

Extraction of experimental urine samples was carried out similar to the method described for development of calibration curves. Briefly, 100 μL of urine from rats dosed with rebamipide was placed in clean glass tubes and spiked with 100 μL IS. Afterwards, 20 μL of 36% hydrochloric acid was added followed by 2 mL of acetonitrile. Solutions were vortex mixed for 1 min, then centrifuged for 20 min at 5000 × *g*. After centrifugation, supernatant was transferred to clean glass tubes and evaporated to dryness then reconstituted in 200 μL mobile phase and placed in clean injection vials for HPLC analysis.

### HPLC system

The HPLC system consisted of an LC020AB solvent delivery system, SIL-20A HT auto-sampler, CBM-20A communication bus, DGU-20A3 vacuum degasser, RF20A fluorescence detector (excitation 320; emission 380), and CTO-20A column oven. The mobile phase used for rebamipide consisted of acetonitrile:water:acetic acid (30:70:5). Rebamipide separation was carried out using a C_18_ (100 × 4.6 mm, 5 μm) analytical column (ACE, Aberdeen, Scotland). Flow rate was set at 0.5 mL/min.

### HPLC analysis

**Fig. 2:**
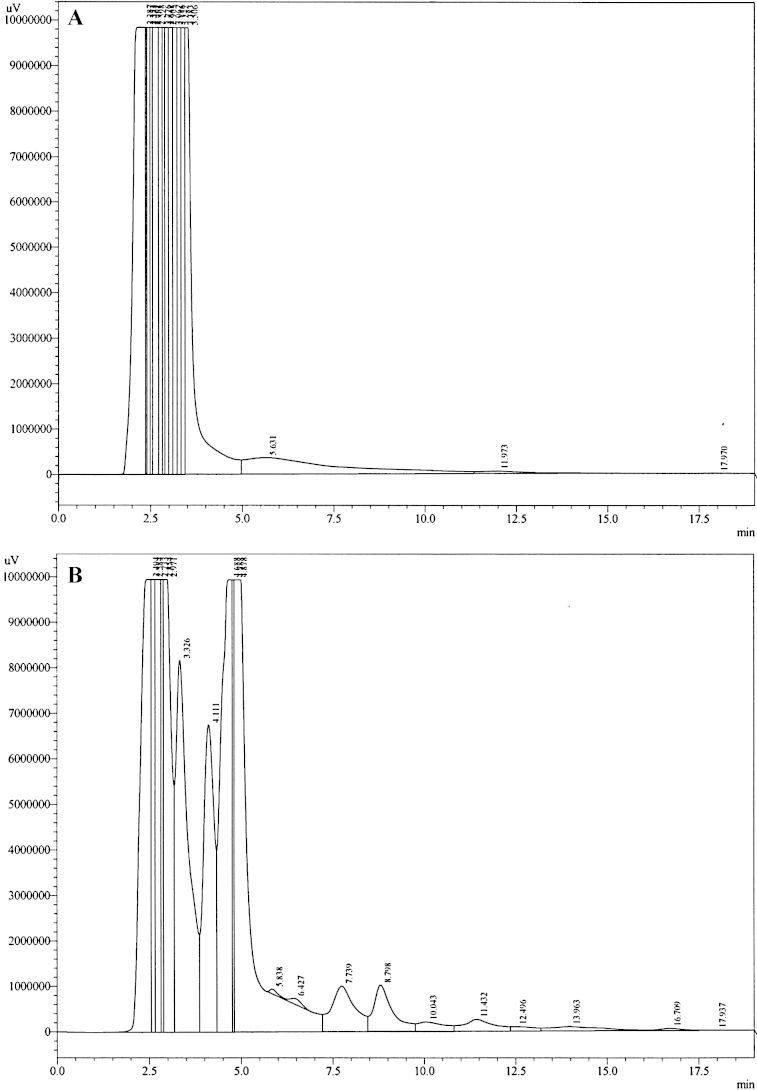
Representative HPLC chromatogram of (A) blank rat urine and (B) rat urine spiked with 100 μL IS.

**Fig. 3:**
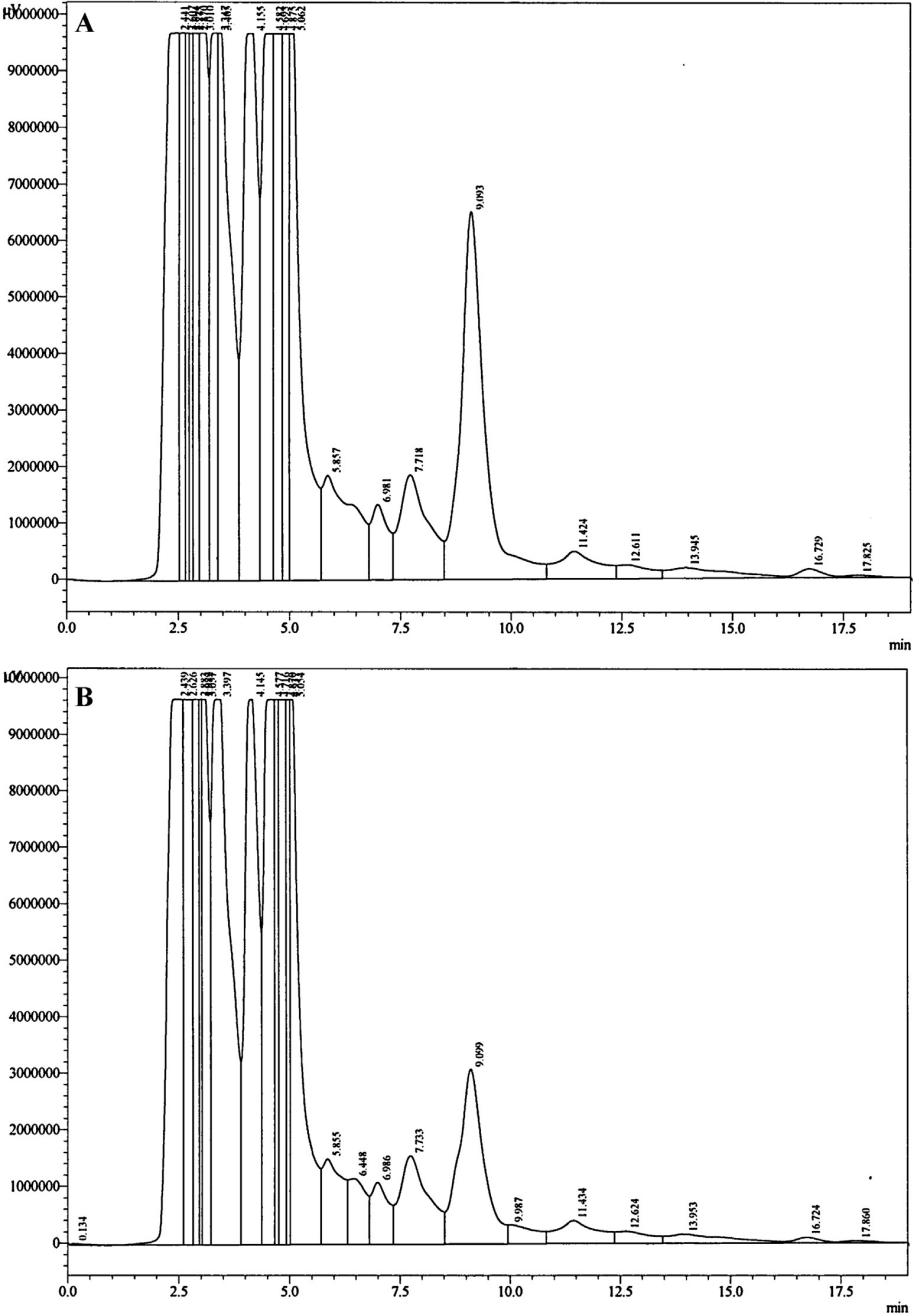
Representative HPLC chromatogram of (A) rat urine spiked with 10,000 ng/mL rebamipide and (B) rat urine spiked with 5000 ng/mL rebamipide.

**Fig. 4:**
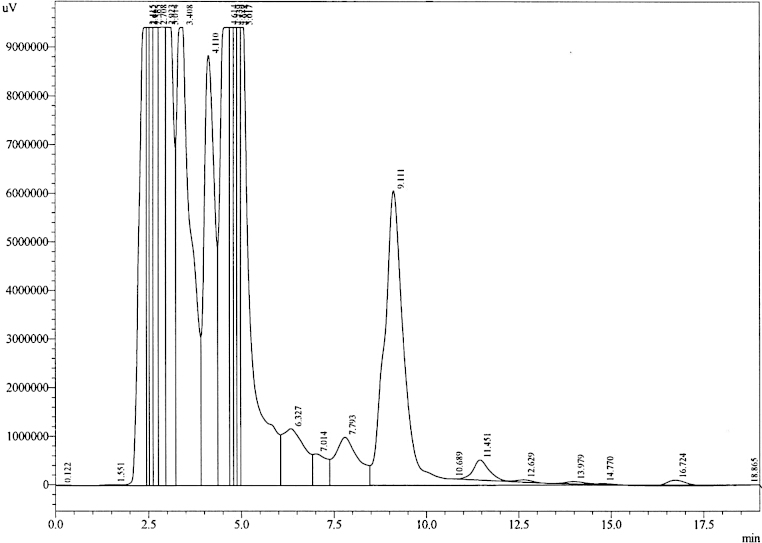
Representative HPLC chromatogram of urine sample obtained from a rat dosed with rebamipide.

HPLC analysis was performed using the Shimadzu LC Solutions software package. Linearity was achieved for both low and high calibration curves by plotting peak area ratios between detected rebamipide and IS against known concentrations of rebamipide. Fig. 1A and B demonstrates the average of three measurements for each concentration. Coefficient of variation was calculated to be 18.9%. Fig. 2A shows the chromatogram of blank rat urine. Elution of IS only samples showed multiple peaks within 4 min time frame, however based on the sensitivity of fluorescence detection, identification of consistent peak areas was achieved at 4.2 min (Fig. 2B). Analysis of calibration samples containing known concentrations of rebamipide demonstrated sufficient detection of rebamipide with peak elution times of 9.1 min (Fig. 3A and B). Analysis of extracted experimental rat samples showed similar elution time for rebamipide at 9.1 min (Fig. 4). As such, rebamipide elution time was determined and concentrations in experimental urine samples were calculated.

## Additional information

### Background information

Various prostaglandins function to regulate and control gut health and gastrointestinal (GI) integrity [6]. Rebamipide acts as a prostaglandin inducer and is often used for prevention and treatment of gastro-duodenal ulcers [1]. As such, studies have been performed looking at rebamipide pharmacokinetics in conjunction with known GI irritants [3,7,8]. These studies often utilize human or animal plasma samples for rebamipide detection and analysis under chromatographic conditions [2,4,9]. Recently, we reported on the pharmacokinetic interactions of rebamipide with select nonsteroidal anti-inflammatory drugs known to promote adverse GI side effects [3]. Our study utilized a previously developed method to determine plasma concentrations of rebamipide in experimental plasma samples [4]. Elucidation of rebamipide elimination processes involved the analysis of urinary concentrations of rebamipide in various treatment samples. Through the modification of methods previously developed for plasma examination of rebamipide we were able to successfully develop a simplified protocol for the analysis of urinary concentrations of rebamipide.
